# Impact of COVID-19 on the mental health of healthcare workers in different regions of the world

**DOI:** 10.1192/bjo.2021.689

**Published:** 2021-06-18

**Authors:** Mahfuja Islam, Philip George, Sindhu Sankaran, Janet Leu Su Hui, Tzun Kit

**Affiliations:** 1 Ashford and St Peter's NHS Foundation Trust; 2 International Medical University; 3 All India Institute of Medical Sciences

## Abstract

**Aims:**

The global health system is facing a serious challenge after the recent outbreak of COVID-19 coronavirus infection which was first identified in Wuhan, China in November 2019 and declared as a pandemic in March 2020 by WHO. There is a wide consensus that this pandemic has negative psychosocial consequences as well as unforeseeable provision of mental health care services and just not on physical health alone. The aim of this research study is to determine the prevalence of psychological distress and to identify the sociodemographic variables with the main attributable factors associated with the psychological distress among healthcare workers and suggestions on how to reduce the impact on the mental health of healthcare workers during the COVID-19 pandemic in different regions of the world.

**Method:**

We performed a cross-sectional study from September-November 2020. We used a self-administered survey tool which was distributed electronically to healthcare workers across the globe. The data were stored on an online database with password protected devices where survey responses were restricted to investigators exclusively.

Data collected were: 1) Socio-demographic data (age, gender, marital status, ethnicity, religion, role in the healthcare, region of practice); 2) Psychological General Well-Being Index (PGWBI) questionnaire which contains 22 standardized items. This is a subjective assessment to score the degree of psychological well-being by focusing on 6 domains: depression; anxiety; positive-well-being; self-control; vitality and general health; 3) Subjective assessment from respondents of the main attributable factors causing psychological distress and suggested methods to help reduce the impact on mental health on health care workers.

**Result:**

Majority out of the 217 respondents were from a younger age group; females and married/domestic partnership, mainly from Western Pacific Region, South East Asian and the African Region. More than half the respondents were moderate-severely psychologically distressed and the three main attributable factors causing psychological distress were: fear of family/friends contracting COVID-19 followed by lack of PPE and discomfort caused by wearing PPE for long hours. Respondents suggested that the distress would be reduced if: more resources were provided in hospital; protocols and guidelines were implemented and counselling facilities with recreational activities were available to frontline workers.

**Conclusion:**

This study showed that the COVID-19 pandemic has affected the mental health of healthcare workers and more support or strategies need to come in place to protect frontline workers at the time of crises.

